# Phosphorylation Dynamics Dominate the Regulated Proteome during Early *Xenopus* Development

**DOI:** 10.1038/s41598-017-15936-y

**Published:** 2017-11-15

**Authors:** Elizabeth H. Peuchen, Olivia F. Cox, Liangliang Sun, Alex S. Hebert, Joshua J. Coon, Matthew M. Champion, Norman J. Dovichi, Paul W. Huber

**Affiliations:** 1 Department of Chemistry and Biochemistry, University of Notre Dame, Notre Dame, IN 46556 USA; 2 Department of Chemistry, Michigan State University, East Lansing, MI 48824 USA; 3 Department of Chemistry, University of Wisconsin, Madison, WI 53706 USA

## Abstract

The earliest stages of animal development are largely controlled by changes in protein phosphorylation mediated by signaling pathways and cyclin-dependent kinases. In order to decipher these complex networks and to discover new aspects of regulation by this post-translational modification, we undertook an analysis of the *X*. *laevis* phosphoproteome at seven developmental stages beginning with stage VI oocytes and ending with two-cell embryos. Concurrent measurement of the proteome and phosphoproteome enabled measurement of phosphosite occupancy as a function of developmental stage. We observed little change in protein expression levels during this period. We detected the expected phosphorylation of MAP kinases, translational regulatory proteins, and subunits of APC/C that validate the accuracy of our measurements. We find that more than half the identified proteins possess multiple sites of phosphorylation that are often clustered, where kinases work together in a hierarchical manner to create stretches of phosphorylated residues, which may be a means to amplify signals or stabilize a particular protein conformation. Conversely, other proteins have opposing sites of phosphorylation that seemingly reflect distinct changes in activity during this developmental timeline.

## Introduction

The path from a fully-grown oocyte arrested in meiosis I, to a fertilizable egg arrested in meiosis II, and onto a diploid zygote, occurs in distinct steps initiated by a steroid hormone in the first instance and sperm entry in the latter. Amphibians have been especially valuable for the identification and characterization of the signaling pathways that mediate these transitions, because the individual steps are easily manipulated. *Xenopus* oocyte maturation and egg fertilization can be performed *in vitro*, generating a large population of synchronized cells amenable to temporal studies. For these reasons, the activities that control these processes such as maturation promoting factor (MPF) and cytostatic factor (CSF) were first identified in frog^1^ and much of our understanding of early animal development comes from work using *Xenopus* as a model organism^2–5^.

Stable isotope labeling has greatly advanced large-scale quantitative proteomic analysis by mass spectrometry (MS)^6,7^. As a result, deep proteome analyses of a number of model organisms of animal development have been reported^8–10^. Several proteomic studies have focused on *Xenopus* eggs, embryos, and blastomeres and have provided unprecedented information regarding the means by which protein expression is regulated during development, and how this regulation impacts processes such as the length of the cell cycle and partitioning of proteins between the nucleus and cytoplasm^11–17^.

While cellular regulation can be achieved through simple control of protein expression, it is clear that post-translational modifications are widely used to regulate protein activity and stability. In particular, signaling pathways involving a panoply of kinases orchestrate the maturation of an animal oocyte to an egg and control the cell cycle after fertilization. For example, phosphorylation coordinates the unmasking of maternal mRNAs, the activation of the anaphase promoting complex (APC/C), and the assembly/disassembly of the nuclear membrane and mitotic spindle. Because a considerable amount of information has been derived from studies using *Xenopus* oocytes and eggs, they provide a logical target for a deeper examination of the vertebrate phosphoproteome during early development. The first examination of the *Xenopus* phosphoproteome identified 1441 phosphorylation sites on 654 proteins^18^ and a subsequent study identified 1738 sites^19^. Together, these two investigations tallied 2636 unique sites.

We have analyzed the *Xenopus* phosphoproteome at seven time points beginning with fully grown stage VI oocytes, through oocyte maturation, and intervals following fertilization. iTRAQ labeling combined with Ti-IMAC enrichment for phosphopeptides enabled us to identify 8974 phosphorylation sites on 5169 different proteins. We combined measurements of the proteome and the phosphoproteome to determine the occupancy change at any individual amino acid position. The concurrent measurement of protein expression and phosphopeptide sites revealed the kinetics of occupancy of 4679 phosphorylation sites across the seven developmental time points. We have captured many of the well-documented phosphorylation events that occur during these developmental stages, which lends confidence in the reliability of this data set. In addition, the data also provide new insights into phosphoproteomics in general.

## Results and Discussion

### Deep proteomic and phosphoproteomic analysis of *X*. *laevis* oocyte maturation and fertilization

**Figure 1:**
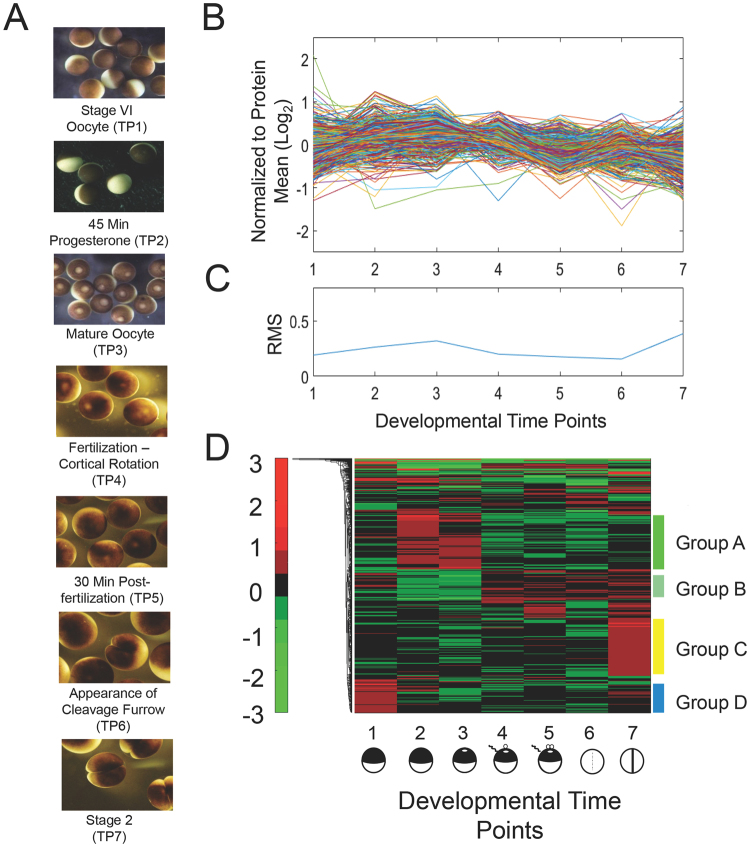
Quantitative changes of the *X*. *laevis* proteome over seven developmental stages. (**A**) Micrographs of *X*. *laevis* at the developmental stages taken for proteomic analyses. (**B**) Individual protein abundance presented on a log_2_ axis across developmental time points. Data are normalized to the mean value of each protein. (**C**) Relative standard deviation (RSD) for all proteins in panel (**B**) at individual experimental time points. (**D**) Heatmap showing the change in protein expression across the developmental time points. The heatmap was generated from the log_2_ normalized data using the default parameters in Matlab. Six groups representing significant trends of protein expression were manually selected for analysis. Proteins with the 15% greatest quantitative change are presented and are considered significant at a FDR of 0.20 using a Benjamini-Hochberg test. Neither clustering nor dendrogram generation was performed along the development stage axis of the heatmaps. These points are defined by the biology of development, and their differences are fixed by that biology.

Four independent 8-plex iTRAQ experiments were performed to determine quantitative changes in the proteome and phosphoproteome of *Xenopus laevis* at seven developmental stages (Fig. 1A). The sequence begins with fully grown, stage VI oocytes arrested in prophase of meiosis I (PI, experimental time point 1), followed by two time points after progesterone-induced maturation to a fertilizable egg. One taken 45 minutes after exposure to hormone (TP2) and the next taken approximately six hours later (TP3) following germinal vesicle breakdown (GVBD). The four remaining time points are subsequent to fertilization: the first (TP4) at cortical rotation (to insure successful fertilization) and then at 30 minutes (TP5). The final two samples were taken just as the cleavage furrow became visible (TP6) and, finally, the fully formed two-cell embryo (TP7). Biological duplicates were analyzed for all time points in technical duplicate. Protein identification and quantitation followed procedures described earlier^17,20^.

Protein samples were digested with trypsin, iTRAQ labeled, and collectively pooled; phosphoproteomic digests were further enriched with Ti-IMAC. Both the proteomic and enriched phosphoproteomic digests were fractionated using high pH reverse phase chromatography and analyzed using nano-UPLC-mass spectrometry. Roughly 2.65 million MS/MS spectra from 30 fractions were acquired for the proteome and 1.21 million MS/MS spectra from eight fractions for the phosphoproteome, totaling over 200 instrument hours between the two experimental conditions. The .raw files were analyze using MaxQuant and the Genome 9.1 database available from Xenbase. Data were filtered with a peptide identification rate of ~99% (peptide-level FDR set at 0.01).

Biological duplicates of the proteome exhibit strong intensity correlation (>0.95 R^2^ value) at equivalent time points. Of the 6428 identified proteins, 4938 were found in both biological samples. A total of 61,041 peptides were identified (Supplementary Table S1). When compared to published experimental data for the stage 1 embryo (TP5 of this study), the Pearson coefficient was >0.79 for five separate experiments^16,17^. Protein copy numbers and protein amounts are highly correlated (Pearson coefficient > 0.95) with the data of Smits *et al*. that used dimethyl labeling^16^.

For the phosphoproteome experiments, biological duplicates and technical duplicates provided reasonable correlation (Pearson coefficient > 0.7) for all time points and when comparing the corrected intensities for both of the biological replicates at identical time points. Of the 5169 identified proteins, 3583 were found in both biological samples (Supplementary Table S2). A total of 15,597 peptides were identified. The phosphoamino acid distribution was 76% serine, 21% threonine, and 3% tyrosine.

Changes in the abundance of a phosphopeptide can have two causes. Protein expression is constant, but the degree of phosphorylation changes. Alternatively, protein expression changes at a constant level of phosphorylation. Of course, both protein abundance and the extent of phosphorylation can simultaneously change. To separate these phenomena, we quantified changes in both the proteome and phosphoproteome^21^. The concurrent measurement of protein abundance and phosphopeptide levels enabled us to determine the absolute occupancy of 4679 phosphorylation sites across the seven developmental time points (Supplementary Table S3).

### Quantitative changes in protein expression

Over the developmental period from oocyte maturation through the first zygotic cleavage, we find that there is not a substantial change in the *Xenopus* proteome (Supplementary Fig. S1). Figure 1B presents a spaghetti plot of the normalized log_2_ intensities for proteins that were observed at all time points; very few proteins showed large deviation from the mean. The relative standard deviation (RSD) in normalized (log_2_) intensity for the population of proteins at each time point is presented in Fig. 1C. Only 486 of the 6428 proteins exhibited a protein expression change greater than 5%. While many of these proteins are involved in general metabolic processes, there are some noteworthy patterns of protein expression that correlate with the biological events that occur during these stages of early development (Fig. 1D, Supplementary Table S4).

**Figure 2:**
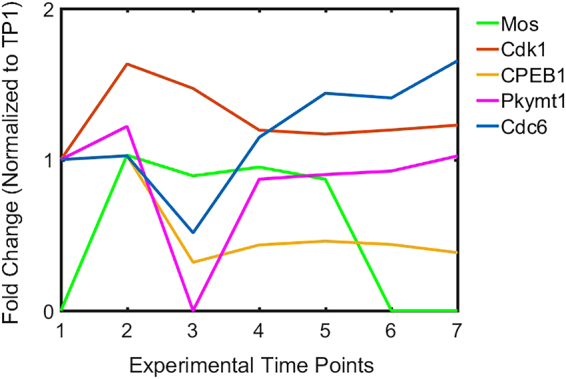
Quantitative changes of proteins that mediate oocyte maturation. Protein abundance normalize to stage VI oocyte are plotted at the seven experimental time points. An average of protein intensity for biological replicates is shown. Standard deviations for each protein are less than 0.2 for all time points. Proteins that were not detected have had their abundance set to zero. Mos was normalized to experimental time point 2.

Pioneering studies by Smith and coworkers determined that there is an approximate two-fold increase in the rate of protein synthesis following progesterone-induced oocyte maturation that, nonetheless, did not detectably change the overall pattern of expression^22^. The expression levels measured here by quantitative mass spectrometry are in agreement with this earlier work. Group A (Fig. 1D) contains proteins that increase during oocyte maturation, but then decline after fertilization. An important member of this group is Mos, the kinase that initiates the MAP kinase pathway that leads to activated M-phase promoting factor (MPF). Mos, which is encoded by masked maternal mRNA, is not detected in stage VI oocytes, but appears 45 min after progesterone treatment, and remains reasonably constant until fertilization when it declines to an undetectable level (Fig. 2). Interestingly, Cdk1 also exhibits an immediate increase of approximately 60% that would enable the formation of Cdk1/Ringo, which is responsible for the initial progesterone-dependent inactivation of Myt1 kinase^23^. In addition, this increase in Cdk1 mirrors that of cyclin B^24^, indicating a coordinated expression of the two MPF subunits (Fig. 2).

MetaCore GO analysis of proteins in Group B, which increase following progesterone treatment, detected enrichment for processes involved in fertilization that can be attributed to the appearance of proteins such as ZP2, ZP3, ZP4, and ZPAX, glycoproteins that confer fertilization competency on the egg, and uroplakin 1b, a component of the complex that is believed to act as the sperm receptor^25,26^. Enriched molecular functions for this group include calcium transport^27,28^, phosphatidylinositol signaling^29,30^, and adenylate cyclase activity^31,32^ that, likewise, are all essential activities for egg activation at fertilization and exit from meiotic arrest. Thus, many of the quantitative increases in the proteome during oocyte maturation manifest the cell’s preparation for the next developmental event, fertilization.

Conversely, the proteins in Group F decline immediately after progesterone treatment (TP2). This group includes proteins involved in the storage and activation of maternal RNA such as ZAR1, a repressor of translation, that regulates the early expression of Mos (prior to GVBD) and late expression of Wee1 (post GVBD). The temporal difference in the translational activity of these mRNAs may result from diminishing levels of ZAR1 and its lower affinity for Mos relative to Wee1 mRNAs^33^. Another member of this group, cytoplasmic polyadenylation element binding protein 1 (CPEB1), acts as a translation repressor and activator. Earlier analyses by western blotting showed an approximate 70% decrease of CPEB1 during oocyte maturation that enables the expression of “late class” mRNAs (*e*.*g*., cyclin B) at or following GVBD (TP3)^34,35^. Over the interval from 45 minutes (TP2) to six hours post-progesterone treatment (TP3), we measure an 80% decrease in CPEB1. CPEB1 rises at fertilization only to drop again at cytokinesis (Fig. 2). Thus, the programmed degradation of CPEB1 needed for oocyte maturation appears to repeat itself during the first cell division cycle. Unexpectedly, Cdc20 (Fizzy), an activator of the APC/C complex, is also found Group F. This decline at GVBD may represent its partial degradation as part of a mechanism that fine tunes APC/C activity that enables release from meiosis I and arrest in metaphase II^5,36^.

Group C contains proteins whose expression increases modestly at fertilization and then remains constant. The proteins in this group are mostly associated with general metabolism and the cytoskeleton.

A second increase in expression occurs upon completion of the first cell division (Group E). Proteins involved in DNA replication, cell-cell contact, and ubiquination are predictably present in this group. Oocytes arrested in PI have lost the ability to enter S-phase, which has been attributed to the absence of Cdc6 in the oocyte that prevents formation of replication initiation complexes, although other activities associated with GVBD could not be excluded^37,38^. In support of this mechanism, we detect a distinct drop in the amount of Cdc6 at GVBD (Fig. 2).

The GO analysis of group E unexpectedly showed functional enrichment of proteins involved in mRNA binding and process enrichment in RNA transport and mRNA processing that can be attributed to the appearance of several splicing factors and RNA helicases. This increased synthesis of proteins involved in mRNA metabolism would support recent evidence for limited, but essential, transcriptional activity prior to the midblastula transition^39^.

### Dynamics of the phosphoproteome during early development

**Figure 3:**
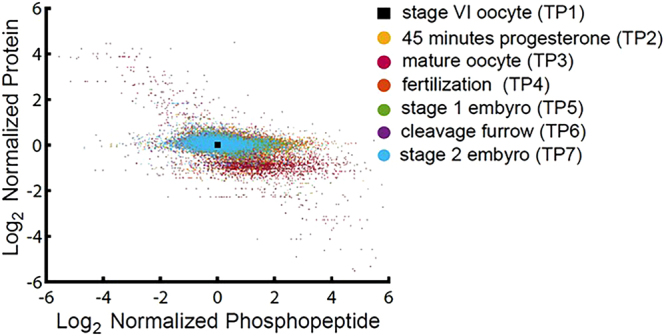
Galaxy plot correlating changes in protein levels with changes in phosphorylation. Individual protein abundances (plotted on the log_2_ axis) normalized to amount present in stage VI oocytes are plotted relative to phosphorylation site occupancy of that protein.

The results of the quantitative proteomic analysis demonstrate that progression from a mature oocyte to an egg and subsequent fertilization to produce a zygote entails the differential expression of a limited number of critical proteins encoded mostly by maternal RNAs, but not a large-scale reprogramming of the proteome. Therefore, the precise temporal regulation of protein activities that underlie the transitions between these developmental stages must rely on well-documented post-translational modifications, especially protein phosphorylation. A galaxy plot of changes in the amount of individual proteins *versus* changes in the amount of phosphorylation at individual sites at each time point relative to stage VI oocytes corroborates this point (Fig. 3 and Supplementary Fig. S2).

**Figure 4:**
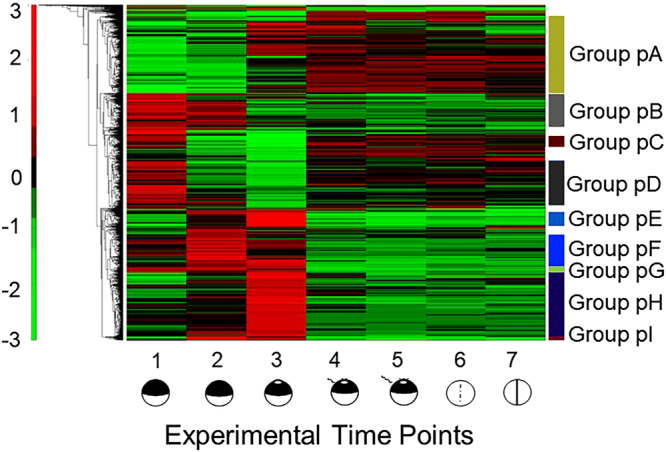
Heatmap displaying changes in absolute occupancy of individual phosphorylation sites. Nine groups were manually selected from the top 25% of sites having the greatest variation in phosphorylation across the seven experimental time points. The heatmap was generated using the MatLab Euclidian algorithm from the log_2_ normalized data. Individual cluster nodes were manually excised for further analysis. All values included are significant at a FDR of 0.15 using a Benjamini-Hochberg test.

The elliptical profiles demonstrate that, in general, phosphorylation is changing to a much greater degree than protein levels. ANOVA analysis^40^ suggests that this differential distribution is significant (α value of 0.05). There are two particularly notable features of this plot. Transition to a mature oocyte exhibits the greatest increase in phosphorylation, yet is the only time point with an appreciable decrease in the amount of several proteins. This result is consistent with an increase in the activities of several kinases, triggered by progesterone, that leads to targeted destruction of proteins involved in masking mRNA as well as ubiquitin-dependent processes needed for release from meiotic arrest. The opposite behavior is seen with the stage 2 (two-cell) embryo, where there is an increase in the amount of protein, but relatively smaller changes in phosphorylation. This behavior is also seen in the hierarchical clustering of changes in phosphoproteins (Fig. 4, Supplemental Fig. S3, Supplemental Table S5). While there are appreciable changes in phosphorylation during oocyte maturation and fertilization, all subsequent time points for the zygote show a much smaller dynamic range.

#### Oocyte maturation

**Figure 5:**
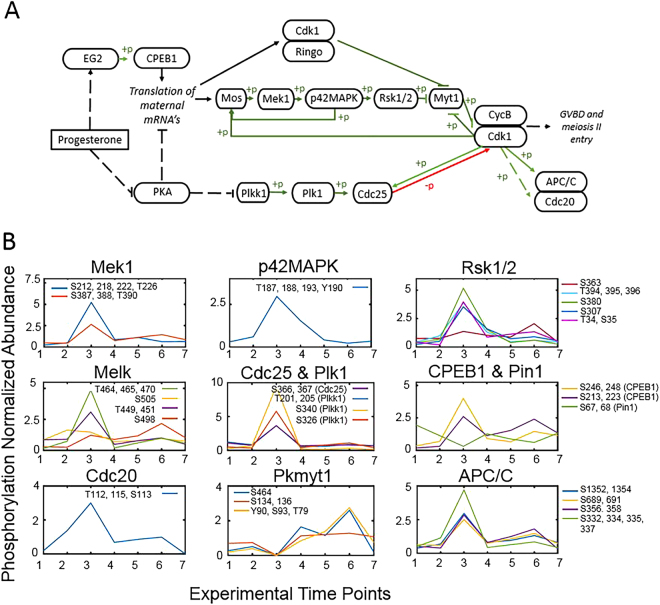
Phosphorylation events during progesterone-dependent oocyte maturation. (**A**) Outline of the major members of the signaling pathway for oocyte maturation initiated by progesterone adapted from^87^. Phosphorylation events are represented in green and dephosphorylation in red with arrows indicating activation and bars indicating repression. Pathways with intermediates not shown are indicated with a broken line. (**B**) The occupancy of individual phosphorylation sites in proteins within the pathway is presented relative to the seven experimental time points. Changes are normalized to the mean of the individual phosphopeptide. Inserts of each panel identify the detected sites of phosphorylation.

Progression from a stage VI oocyte (TP1) arrested in PI to a mature egg arrested in MII (TP3) is orchestrated by sequential unmasking of maternal mRNAs and the resulting activation of multiple kinases, producing an exceptionally dynamic phosphoproteome that coordinates this complex transition^41^. By the end of oogenesis, a small amount of inactive MPF has accumulated. PI arrest is maintained by phosphorylation of the Cdk1 subunit of MPF by Myt1 kinase. Progesterone stimulation ultimately leads to the dephosphorylation of Cdk1 by Cdc25 phosphatase to produce active MPF and progression to metaphase II arrest (Fig. 5A). The earliest steps in the activation of MPF can be traced to the translational derepression of Ringo mRNA. The expression of RINGO/CDK acts on two levels: direct phosphorylation and inactivation of Myt1^23^ and phosphorylation of Musashi that, in turn, allows expression of Mos and activation of the MAP kinase pathway that also targets Myt1^42^. In parallel, Plk1 activates Cdc25 by phosphorylation. The combined inhibition of Myt1 and activation of Cdc25 leads to the burst of MPF activity that is then maintained by various amplification loops as well as sustained expression of Mos^41^.

Our analysis has captured several of the temporal changes in phosphorylation that control cell cycle progression following progesterone stimulation (Fig. 5B). Groups pE and pH contain proteins that exhibit a marked increase in phosphorylation at GVBD and include: Plk1, Mek1, p42MAPK, Rsk1/2, Cdc25, MELK (maternal embryonic leucine zipper kinase), Cdc20, and Apc1 (a subunit of the anaphase-promoting complex/cyclosome). The phosphorylation of Plk1 results from the decrease in intracellular cAMP upon progesterone stimulation; the activated Plk1 then phosphorylates Cdc25. In parallel, *de novo* synthesis of Mos initiates the MAP kinase pathway made up of Mek1, p42MAPK, Rsk1/2, and Myt1. We detect highly phosphorylated forms of all these proteins at GVBD (TP3) except for Myt1. Inactivation of Myt1 is the result of two kinase activities with initial phosphorylation by RINGO/CDK that facilitates subsequent recruitment and phosphorylation by Rsk1/2^43^. We did not detect phosphopeptides corresponding to the target sites of either kinase, accounting for the apparent absence of phosphorylation of Myt1 at GVBD. However, we did detect phosphorylation of the protein after fertilization at sites in the N-terminal kinase domain and the C-terminal Cdk1/cyclin B interaction domain; none of these sites is predicted to be a target of cyclin-dependent kinases. Apparently, phosphorylation of Myt1 in the zygote is used for a distinctly different purpose compared with its role in regulating progression through meiosis I.

Entry into anaphase requires the ubiquitin-dependent destruction of the cyclins and securin that is mediated by APC/C. Cdc20 is a positive activator of the APC/C that is negatively regulated through phosphorylation by Bub1. The single Cdc20 phosphopeptide that we detect at TP3 does not correspond to any of the reported sites phosphorylated by Bub1^44^, but instead has a putative consensus sequence for Cdk1 embedded with one for CK2, strongly suggesting that phosphorylation at this site is not involved in negative regulation of Cdc20, but rather its activation. We also detect phosphorylation of the Apc1 subunit of APC/C at TP3. It was recently shown that phosphorylation at multiple CDK sites in the loop domain of Apc1 is required for binding of Cdc20^36^. We detect phosphorylation at several of these sites as well as at other positions. We pose that phosphorylation of Cdc20 and APC/C by Cdk1 is required for activation of the complex and exit from meiosis I.

As expected, groups pE and pH also include proteins involved in the regulated translation of masked transcripts (eIF4G, cytoplasmic poly(A) binding protein, eIF3a, eIF4E binding protein, ELAV1). We also find an appreciable number of nucleoporins in these groups. Phosphorylation is required for the disassembly of the nuclear pore complex and, hence, GVBD. Many of these proteins are targeted by kinases that are activated upon oocyte maturation, especially Cdk1, but also PKC, Plk1, and Aurora A (Eg2)^45^. Proteins in these groups are associated with cell cycle processes, chromosome organization, nuclear envelope organization, RNA/nucleic acid binding, cytoskeletal interactions, and nuclear pore structure. GO results also show enrichment for proteins involved in cell-cell adhesion; indeed, expression of some cadherins from maternal mRNA during *Xenopus* oocyte maturation has been reported^46,47^.

Group pF contains proteins that exhibit an increase in phosphorylation at 45 min (TP2) and includes CPEB1, an early target of Aurora A. Phosphorylation of S174 enables the interaction of CPEB1 with CPSF, which triggers cytoplasmic polyadenylation of early maternal mRNA^48^. Subsequent phosphorylation by Cdk1 and Plk1 marks CPEB1 for proteolytic degradation^35,49^. We did not detect a phosphopeptide corresponding to S174, but did identify four other sites of phosphorylation. Two residues, S210 and S223, show some phosphorylation at 45 minutes that reach a maximum after GVBD, while T246 and S248 exhibit a marked increase only after GVBD. It has been demonstrated that Cdk1 targets S210 and S248, with phosphorylation of the former site required for high affinity binding of the prolyl isomerase, Pin1, whose action is required for the ubiquitin-dependent destruction of CPEB1^50^. While phosphorylation at S223 and T246 has not been previously reported, deletion of the region encompassing residues 211 to 290 prevents association of Pin1 with CPEB1^51^. Pin1 (group pD) is found in an inactive form associated with CPEB1 in oocytes. Upon progesterone stimulation, Pin1 is rapidly converted into an enzymatically active form by dephosphorylation at S68 (S71 in human Pin1)^51,52^. We detect a marked dephosphorylation of S68 at 45 min that continues through GVBD (Fig. 5B). Phosphorylation of Pin1 is restored following GVBD (TP4) and then declines after fertilization as the egg progresses through its first mitotic cycle. Finally, phosphorylation is reestablished immediately after cell division (TP7). This striking cyclic behavior suggests that Pin1 phosphorylation/dephosphorylation is synchronized with the cell cycle, with its activity fully expressed during M phase. In addition, we have detected unreported phosphorylation at the adjacent serine residue (S67) that exhibits the same cyclical behavior.

GO analysis of Group pF detected enrichment for miRNA biogenesis and regulation by miRNA, which is particularly notable in light of recent studies that determined CPEB1 interacts with miRNA complexes through Ago2 to temporally regulate the translation of cyclin E1 mRNA during oocyte maturation^53^. In addition, miRNA complexes are required to maintain the level of oocyte Myt1 and, thus, arrest in PI^54^. The GO analysis is not only consistent with emerging evidence for translational control by miRNA complexes during oocyte maturation, but also indicates that this mechanism, which may be more widespread than previously realized, is regulated by phosphorylation.

Groups pG and pI also contain proteins whose phosphorylation increases immediately after progesterone treatment, but with somewhat different kinetics at subsequent time points. The former group shows GO enrichment in RNA/mRNA metabolic processes and RNA-directed RNA polymerase activity, which may be related to the miRNA activities described above. Also included in these groups is MARCKS, a cellular substrate for protein kinase C, which becomes rapidly activated upon hormone treatment and controls a remodeling of the cytoplasm necessary for fertilization competency^55^ and HURP, a protein involved in spindle formation that is associated with Aurora A^56^.

Conversely, groups pB and pE are characterized by dephosphorylation immediately following progesterone treatment. GO analysis shows functional enrichment in both groups for RNA binding, poly(A) binding, translation initiation factor activity, and cell-cell adhesion. These groups includes proteins involved in the expression of masked mRNAs, including Pin1 (discussed above), eIF4G-1, which appears to play a role in the translation of maternal mRNAs that code for proteins essential for the completion of meiosis I^57^, and symplekin, which is an essential component of the complex that directs cytoplasmic polyadenylation of maternal mRNAs^58^.

Fragile X mental retardation protein (FMRP) (group pD) is highly expressed in *Xenopus* oocytes^59^. The protein can repress translation by a direct interaction with ribosomes^60^. Perhaps of greater relevance in this case, FMRP in mouse neurons forms a complex with Ago2:miRNA to repress translation of specific mRNAs, which is reversed by dephosphorylation^61^. A subset of miRNAs is enriched in *Xenopus* oocytes, but is nearly absent in eggs^62,63^. Two (T505 and S506) out of four phosphorylated residues detected in oocyte FMRP1 show a marked decline immediately after progesterone treatment, which would be consistent with a role for miRNA-directed translational repression of maternal mRNAs that become activated upon oocyte maturation through a mechanism analogous to that in neurons.

Proteins in group pB are phosphorylated in stage VI oocytes (TP1) and do not immediately change upon progesterone treatment (TP2), but are dephosphorylated by GVBD (TP3). GO molecular function enrichment includes RNA, poly(A), and nucleic acid binding, while biological process enrichment includes cell cycle and protein dephosphorylation. The most noteworthy member of this group is Cdk1 whose transient dephosphorylation enables MPF to promote progression into meiosis II. The measured dephosphorylation of Mapk12 (p38γ/SAPK3) likely represents its inactivation as an independent kinase that targets Cdc25 and has been proposed to be a pathway to oocyte maturation that is complementary to the canonical MAP kinases^64^. Similarly, another member of this group, protein kinase Cδ has been reported to induce meiotic maturation when injected into oocytes^65^. The FRGY proteins are part of the storage RNP complexes that form on maternal mRNAs and are highly phosphorylated in oocytes on multiple CK2 sites^66^. We detect 10 sites of phosphorylation that match those identified earlier in oocytes and speculate that the observed dephosphorylation of this family is part of the unmasking process.

#### Fertilization

The marked increase in phosphorylation of proteins at GVBD (TP3) in groups pE and pH is essentially reversed at fertilization (TP4). These groups represent approximately 400 proteins and contain members of the pathways that lead to GVBD and sustain arrest of the egg at meiosis II (*e*.*g*., MEK1, Rsk1/2, Plk1, Plk3, Cdc25, Cdc20, APC subunit 1). Several of these cell cycle associated proteins become phosphorylated again during mitotic M phase (TP6). On the other hand, other members of these groups are associated with translational activation of maternal mRNA (*e*.*g*., CPEB1, eIF4G2, eIF3a, eIF4E binding protein, ELAV1) and these generally remain unphosphorylated after fertilization.

Several proteins in groups pC and pD, which exhibit dephosphorylation immediately after exposure to progesterone (TP2), become phosphorylated again at various times following fertilization. GO analysis detects some enrichment for nucleic acid binding activity, DNA metabolism, cell adhesion, mRNA metabolism, cell cycle, cytoskeletal organization and organelle organization. However, many of the proteins in these two groups are involved in diverse metabolic processes that presumably reflect the energetic demands of the approaching cell division.

The majority of proteins that comprise group pA show an increase in phosphorylation at various times following fertilization, although for a clear subset of this group, this increase has occurred at GVBD. Predominant GO process enrichment for this group includes various aspects of the (mitotic) cell cycle. An example of proteins that show cyclic phosphorylation are lamins B1 and B3, which exhibit maxima at GVBD (TP3) and again during formation of the cleavage furrow (TP6). The sites detected here are mostly targets of CDK1 and phosphorylation at these positions is required for disassembly of lamin filaments during mitosis^67^. Relatedly, CLASP1 is a multifunctional protein that is directed to the spindle midzone and kinetochores and plays an essential role in microtubule polymerization and bundling during mitosis^68^. CLASP1 is phosphorylated at many sites in a complex temporal pattern that seemingly reflects its dynamic interaction network. There are sites that exhibit a mutually exclusive periodicity that reflect its distinct roles. Maximum phosphorylation of S1227 and S1231 during M phase (TP3 and TP5/6) seemingly reflect its activity in spindle organization, whereas several residues in the amino terminal half of the protein, which show greatest phosphorylation during cortical rotation and formation of the cleavage furrow (TP4 and TP6, respectively), more likely reflect the role of CLASP1 in polymerization of noncentrosomal microtubules. CLASP1 is typical of several proteins in the database that show opposing sites of phosphorylation over the time interval studied here, which most certainly reflects regulated changes in protein activity at particular developmental stages. Indeed, this rapid interconversion, which must occur within the unusually short cell cycle of a cleavage stage embryo, could only be accomplished by reversible post-translational modifications such as phosphorylation.

GO functional enrichment in group pA includes RNA/nucleic acid binding, translation initiation, and several aspects of cell-cell adhesion that likely derive from sperm-egg fusion. While the range of biological function increases in this group compared to others, there remain examples of cell cycle control through phosphorylation such as MELK, a target of MPF and a possible regulator of the Cdc25 phosphatase^69^. The pre-replicative complex protein, MCM2, also falls into group pA. We detect phosphorylation at CDK and CK2 sites that have also been identified in human MCM2^38,70^. Phosphorylation of MCM2 by cyclin E/Cdk2 is required for assembly of the pre-replication complex and cell cycle reentry^71^. Thus, the dephosphorylated form of MCM2 in mature oocytes, along with the low levels of Cdc6, likely contributes to the inability to initiate replication.

A remarkable number of proteins in group pA are involved in spindle assembly. PCM1 is required for radial organization and anchoring of microtubules to the centrosome^72^. MAP4/p220 (microtubule associated protein 4) is extensively phosphorylated at TP3 and TP6 consistent with a role in determining meiotic and mitotic spindle assembly^73^. Mutation of sites phosphorylated by MPF alters MAP4 affinity for microtubules resulting in compromised chromosome movement^74^. While we detect phosphorylation of MAP4 at MPF and MAP kinase sites reported earlier^74^, there is a unexpected variety of additional sites that follow the same temporal pattern, including target sites for ATM, DNA-PK, CK1, and several sites for CK2. Similarly, another member of group pA, INCENP, is a component of the chromosomal passenger complex that interacts with CLASP1 and is required for spindle assembly. Plk3, unlike Plk1, has functions beyond cell cycle regulation. Although there have been no reports of phosphorylation of *Xenopus* Plk3, the modification has been detected in the mammalian cells and connected to a variety of processes: cell cycle progression, DNA damage, mitotic spindle disruption, and stress responses^75,76^.

GO analysis of group pA detected considerable enrichment of processes involved in intracellular/organelle organization that can be accounted for by the large number of proteins involved in assembly of the mitotic spindle and chromosome separation, as well as the phosphorylation of several proteins involved in nucleolar (*e*.*g*., nucleolin, Ki-67, nopp130, nop132) and nuclear membrane (*e*.*g*., lamins b1 and b3) structure.

### Proline-directed kinases account for the majority of changes in phosphorylation

**Figure 6:**
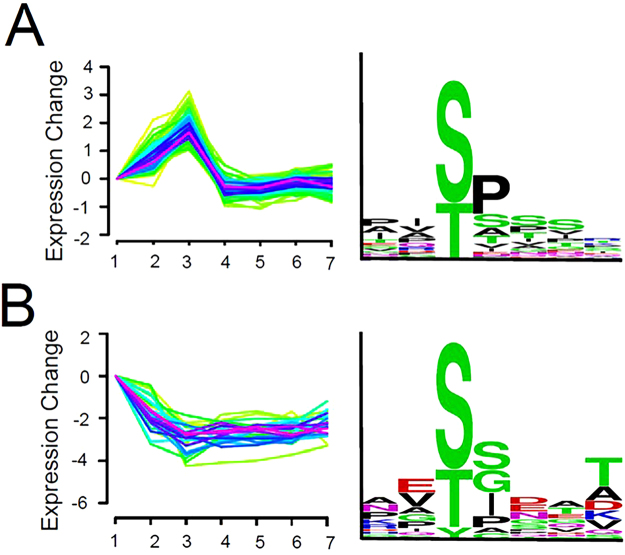
Consensus phosphorylation sites. GproX was used for unsupervised and unstandardized clustering of all identified phosphopeptide sequences. Upper and lower threshold log_2_ values of 0.26 and −0.32 were used, corresponding to ratios of 1.2 and 0.8, respectively. This generated 22 unique clusters according to changes in phosphorylation as a function of developmental time. Expression profile and corresponding consensus sequences generated using WebLogo for (**A**) cluster 20 and (**B**) cluster 21.

Unsupervised clustering of phosphopeptides generated 22 groups with similar temporal patterns of phosphorylation (Supplemental Fig. S4, Supplemental Table S6). Consensus sequences (N_2_S/TN_4_) were generated for each group in an effort to identify kinase activities that predominate during the different developmental stages. As might be expected, the vast majority of consensus sequences have a proline residue immediately flanking the phosphoamino acid and frequently at the −2 position as well (Fig. 6A). The cyclin dependent kinases, MEK1 and p42MAPK, all target these sequences and clearly account for the bulk of detectable protein phosphorylation during oocyte maturation as well as the first mitotic cell cycle.

A considerable number of sites predicted to be phosphorylated by CK2 were also identified. In many cases it appears that initial phosphorylation by a proline-directed kinase then created a site that was subsequently phosphorylated by CK2. Indeed, CK2 is one of just four known kinases whose recognition site can be created through a mechanism known as “hierarchical” or “primed” phosphorylation^77^. This phenomenon accounts for the ubiquitous phosphoserine stretches that occur throughout the human phosphoproteome^78^ that we have also detected in this study.

**Figure 7:**
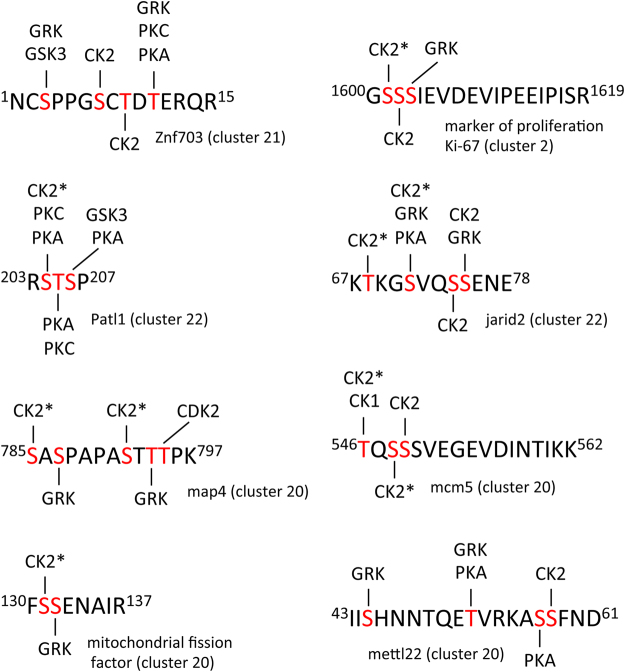
Clustered sites of phosphorylation. A selection of multiple-phosphorylated peptides is presented. Phosphorylation sites indicated in red had a minimum fractional occupancy of >0.1. Predicted kinase substrate sites were identified using PhosphoMotif Finder^86^. GRK (G protein-coupled receptor kinase), GSK3 (glycogen synthase kinase 3), CK2 (casein kinase 2), PKC (protein kinase C), PKA (protein kinase A), CDK2 (cyclin-dependent kinase 2), CK1 (casein kinase 1). CK2* indicates that phosphorylation at this site requires prior phosphorylation of a proximal priming site.

The majority of clusters in which dephosphorylation occurs after progesterone treatment are not dominated by proline-directed kinase sequences; rather, as a group they are enriched in consensus sequences for CK1, PKA, and especially CK2 (Fig. 6B). In many instances, CK1 and CK2 appear to have functioned as priming kinases. In addition, consensus sequences for glycogen synthase kinase 3 (GSK3) occur in several proteins found in these clusters. GSK3, as a member of several disparate signaling pathways, phosphorylates many proteins, often in combination with CK1 and/or PKA (Fig. 7)^79^. Notably, GSK3 activity contributes to PI arrest and its inactivation in response to progesterone is necessary for oocyte maturation^80^. Upon progesterone treatment, decreased level of cAMP will reduce the activity of phosphorylase kinase that, combined with the inactivation of GSK3, should result in dephosphorylation of glycogen synthase. Indeed, we detect loss of glycogen synthase 1 phosphorylation at target sites for phosphorylase kinase, GSK3, PKA and CK1.

Another consensus sequence that occurs frequently is that for G protein-coupled receptor kinase (GRK). This observation is significant because prophase arrest requires constitutive G protein signaling and GRK3 has been implicated specifically in *Xenopus* oocyte maturation^81^. Phosphorylation of the receptor and subsequent binding of β-arrestin appears to be a common mechanism of desensitization. However, GRKs are known to have targets beyond receptor proteins, indicating their activity is not necessarily limited to just the immediate signaling pathway^82^. Additional targets of the GRK could serve as a means to amplify the signal of the agonist allowing for a more global and rapid response.

Our temporal analysis of the *Xenopus* phosphoproteome has detected distinct quantitative changes in phosphorylation upon oocyte maturation that correlate exceptionally well with the decline of kinase activities that maintain the cell at PI arrest (PKA, GSK3) and with the corresponding activation of MAPK and cyclin dependent kinases that control completion of meiosis I and arrest at meiosis II. While these are the principle regulatory kinases, it is clear that general kinases such as CK1 and CK2 play an essential supporting role that either amplifies or stabilizes the effect of the initial phosphorylation event (Fig. 7).

## Conclusions

A fully-grown stage VI oocyte has accumulated most of the proteins that will be needed for fertilization and progression through the first mitotic cell cycle. The dormant or masked mRNAs of the immature oocyte that become activated upon progesterone-dependent maturation to an egg only modestly change the proteome. A notable exception is the synthesis of proteins necessary for fertilization. The vast majority of phosphorylation that occurs during egg formation can be attributed to proline-directed kinases from the MAPK pathway or cyclin-dependent kinases. However, we find that a considerable number of proteins possess multiple site of phosphorylation that are often clustered, which is consistent with recent examples in which GSK3 and CK1 work together in a hierarchical manner to create stretches of phosphorylated residues^77^. Examination of phosphopeptides identified here suggests that proline-directed kinases work with CK2, CK1, and possibly PKA in a similar fashion. This hyperphosphorylation may be a means to amplify signals or, relatedly, stabilize a particular protein conformation. While GO analysis yielded expected enrichments in molecular function and biological process related to translational control, cell-cycle regulation, and spindle organization, those related to regulation by miRNA were unexpected^83^. Thus, recent reports of translational control of maternal mRNAs by miRNAs may be more widespread and, like cytoplasmic polyadenylation, regulated by phosphorylation^53,54^. The excellent agreement between measurements made in this study with well-documented pathways that control oocyte maturation and the mitotic cell cycle engender confidence in the phosphoproteome data and its value for investigations into other processes controlled by this post-translational modification.

## Methods

### Materials

*Xenopus laevis* animals were purchased from Nasco (Fort Atkinson, WI USA). All animal procedures were performed according to protocols approved by the University of Notre Dame Institutional Animal Care and Use Committee. Complete, mini protease inhibitor cocktail and phospho-stop inhibitors, provided in EASYpacks, were purchased from Roche Diagnostics (Indianapolis, IN USA). Human Chorionic Gonadotropin (HCG), bovine pancreas TPCK-treated trypsin, progesterone, 0.5 M triethylammonium bicarbonate buffer, pH 8.5–8.6 (dissolution buffer for ITRAQ labeling), and cysteine were purchased from Sigma-Aldrich (St. Louis, MO USA). Pierce C-18 Spin Columns, and Pierce BCA Protein Assays were purchased from Thermo Scientific (Marietta, OH USA). Sep-Pak Vac (1 cc, 100 mg and 0.5 cc, 50 mg) C-18 Cartridges were purchased from Waters (Ireland). Centrifugal Filter Units with a 30,000 MWCO and Ziptips were purchased from Millipore (Carrigtwohill, CO USA). iTRAQ Reagent 8 Plex Kit and labeling reagent was purchased from Sciex (Framingham, MA USA).

### Oocyte and embryo collection and culture

A sample of ovary tissue was surgically removed, placed in OR2 buffer and oocytes were manually defolliculated. Stage VI oocytes were selected and maturation was induced by incubation at 18 °C with 10 µg/mL progesterone in OR2 buffer. For egg collection, female *X*. *laevis* were injected with 600 units of HCG 12–15 hours prior to spawning; testes were isolated from anesthetized male frogs. Eggs and minced testes were combined in a total volume of 2 to 5 ml 1/3 MMR (Marc’s Modified Ringers) and incubated for 10 minutes. The sample was then flooded with 1/3MMR and incubated for another 20 minutes. Fertilized eggs were washed with 2% L-cysteine for 4 minutes to remove the jelly coat. Embryos were then allowed to develop at ambient temperature. All samples were snap frozen in liquid nitrogen immediately following collection to preserve the experimental time point.

### Protein preparation

Each sample was suspended in 600 μL NP40 buffer (containing phospho-stop and protease inhibitor) and processed as described previously^84^. Samples were prepared in biological duplicate.

### ITRAQ labeling

Each protein sample (100 µg) was labeled according to the manufacturer’s protocols.

### Proteome fractionation

The sample complexity was reduced using high pH reversed phase fractionation prior to mass spectrometry analysis which has been described^84^.

### Proteomic UPLC-ESI-MS/MS analysis

A nanoACQUITY UltraPerformance LC (UPLC^©^) system (Waters, Milford, MA USA) was used for peptide separation. Buffer A (0.1% FA in water) and buffer B (0.1% FA in ACN) were used as mobile phases for gradient separation. Peptides were automatically loaded onto a commercial C18 reverse phase column (Waters, 100 µm ID, 100 mm, 1.7 mm particle, BEH130C18, column temperature 40 °C) with 2% buffer B for 10 minutes at a flow rate of 1.00 µL/ min, followed by a 4-step gradient separation, 1 min from 2% to 8%, 87 minutes to 30% B, 1 minute to 80% B, and maintained at 80% B for the next 10 minutes. The column was then equilibrated for 10 minutes with 2% B before analysis of the next sample. The eluted peptides from the C18 column were pumped through a capillary tip for electrospray, and analyzed by a Q-Exactive™ HF mass spectrometer (Thermo Fisher Scientific). For each sample, approximately 2 μg of peptide was analyzed per run. Electrospray voltage was 2.0 kV, and the ion transfer tube temperature was 280 °C. The S-Lens RF level was 60.00. Data acquisition was programmed in data-dependent acquisition (DDA) mode. For analysis using the Q-Exactive™ HF, instrument settings included: a top 12 method, full MS scans were acquired in Orbitrap mass analyzer over 350–1500 m/z range with a resolution of 60,000, and the number of micro scans set to 1. Automatic gain control (AGC) target value was 3.00 E + 06, the maximum injection time was 30 ms. For MS/MS scans, the twelve most intense peaks with charge state ≥2 and <6 were sequentially isolated and further fragmented in the higher-energy-collisional-dissociation (HCD) cell following one full MS scan. The normalized collision energy was 33%, and MS/MS spectra were acquired in the Orbitrap mass analyzer with resolution 30,000. The first fixed mass was 100.0. The number of micro scans was 1 and the ion selection threshold was 1.0 E + 05 counts.

### Phosphorylation enrichment

Sample preparation was scaled to prepare 500 µg for each channel. A 50 µL aliquot of Ti-IMAC was used for each mg of labeled protein. Beads were washed 3 times with 80% ACN + 6% TFA. Sample was then suspended in 80% ACN + 6% TFA and combined with the beads. The beaded sample was vortexed for 20 minutes at ambient temperature. Beads were washed 3 times with 80% ACN + 6% TFA three times before being washed 80% ACN and 80% ACN + 0.5 M glycolic acid respectfully. The sample was then eluted two times into a new tube of 50% ACN + 1% ammonium hydroxide. Samples were then lyophilized and reconstituted in 0.2% FA at ~1 µg/µL, before being placed on the UPLC-MS/MS system.

### Phosphoproteome fractionation

Samples were fractionated on a Thermo Ultimate 3000 at a flow rate of 0.5 mL/min. Sample was loaded onto a column (3 × 150, 1.7 µm, BEHC18, column temperature 65 °C). The mobile phase gradient was generated using buffer A (10 mM ammonium formate, pH 10) and buffer B (80% MeOH in 10 mM ammonium formate, pH 10). The sample was loaded onto the column followed by a 21-minute wash at 0% B, and then separated by a 3-step, 15-minute gradient, at a flow rate of 0.5 mL/min. 2 minutes at 0 to 25% B, followed by 8 minutes for 25–75% B, 1 minute of 75–100% B, and then maintained for 2.4 minutes at 100% B before re-equilibration at 0% B for remainder of the 15-minute gradient. Eluate from 3–14 minutes was collected at 41 second intervals and then every 9^th^ sample was combined (*i*.*e*., sample 1 added to 9, 2 to 10, *etc*.) to yield 8 total samples. Samples were then dried down and reconstituted in 0.2% FA before being placed on the MS.

### Phosphoproteomics UPLC-ESI-MS/MS analysis

A Thermo Fisher RPLC nano system was used for phospho-peptide separation. Buffer A (0.2% FA in water) and buffer B (80% ACN in 0.2% FA) were used as mobile phases for gradient separation. Peptides were automatically loaded onto a commercial C18 reverse phase column (Waters 75 µm × 25 cm, BEHC18, column temperature of 65 °C) with 0–4% buffer B for 11 minutes at a flow rate of 0.375 µL/min, followed by a 2-step gradient separation, 64 min from 4% to 55%, 1 minute from 55–100% B, and then maintained at 100% for the next 4 minutes. The column was then equilibrated for 10 minutes with 2% B before analysis of the next sample. The eluted peptides from the C18 column were pumped through an integrated capillary emitter for electrospray, and analyzed by an Orbitrap Fusion Lumos mass spectrometer (Thermo Fisher Scientific). For each sample, a 4 µL injection of peptide was placed on the column per run. Electrospray voltage was set at 2.1 kV and the ion transfer tube temperature was 280 °C. The S-Lens RF level was 20. Data acquisition was programmed in data-dependent acquisition (DDA) mode. For analysis using the Fusion, instrument settings included: a top 2 seconds, full MS scans were acquired in Orbitrap mass analyzer over 350–1500 m/z range with a resolution of 60,000, and the number of micro scans set to 1. The automatic gain control (AGC) target value was 1.0E6, the maximum injection time was 100 ms. For MS/MS scans, the most intense peaks with charge state ≥2 and <6 were sequentially isolated and further fragmented in the higher-energy-collisional-dissociation (HCD) cell following one full MS scan. The normalized collision energy was 35%, and MS/MS spectra were acquired in the Orbitrap mass analyzer with resolution 30,000 in centroid mode. The first fixed mass was 100.0. The number of micro scans was 1 and the ion selection threshold was 2.0 E + 05 counts. Peptide match and excludes isotopes were turned on. A maximum injection time of 118 ms was used for MS/MS collection.

### Data searching

Raw files were searched using MaxQuant 1.5.5.1 and the Genome 9 database (downloaded 31 October 2016 from Xenbase) using the default settings for deep proteome analysis. Carbamylation was set as a fixed modification. Variable modifications for the protein analysis included: Acetyl (K), Acetyl (Protein N-term), Deamidation (NQ), and Oxidation (M). For phosphoproteomic analysis the variable modifications used were: Acetyl (K), Acetyl (Protein N-term), Deamidation (NQ), Gln- > pyro-Glu;GlyGly (K), Oxidation (M) and Phospho (STY). A maximum of two missed cleavages was allowed. The minimum charge of the peptide was set to 2 and the max charge was set to 7. The maximum number of modifications per peptide was set to 8, and the maximum mass of a peptide was set to 4000 Da. A minimum of 1 unique peptide was required for protein identification. The false discovery rate (FDR) was set to 0.01 on peptide and protein levels.

### Data analysis

Data was corrected for each iTRAQ channel and normalized according to the mean of the individual protein across all 7 time points for both proteins and phosphorylation sites. To be included in the analysis, the protein or phosphorylation site was required to have an intensity value for analysis in our experiment.

Histograms, heatmaps, clustering, and individual graphs were generated in Matlab using default algorithms and the mean intensity of the protein or site. Protein lists for individual groups derived from the heatmaps were uploaded into the MetaCore^TM^ software suite and subjected to analysis with the Gene Ontology (GeneGo) algorithms. Two GO functional ontologies, biological processes and molecular function, were generated using GO Term classification software CateGOrizer (v. 3.218).

For the clusters analyzed for consensus sequence homology, GProx was used to organize clusters with the following traits: no reference, no standard, 0.2 membership requirement, and threshold of −0.32–0.26 (equating to a 0.8–1.2-fold change minimum)^85^. Consensus sequences were aligned by the active phosphorylation site and generated using http://weblogo.berkeley.edu/logo.cgi. PhosphoMotif Finder was used to identify phosphorylation motifs within the sequence of phosphopeptides^86^.

### Data availability

All raw files have been uploaded to the MassIVE database, including, Phospho enriched, proteome data, MaxQuant Processed files and MaxQuant search parameters (MQpar) (XML) at the following exchange ftp://MSV000081416@massive.ucsd.edu.

## Electronic supplementary material

Supplementary Figures

Dataset S1

Dataset S2

Dataset S3

Dataset S4

Dataset S5

Dataset S6
