# Author Correction: Phosphorylation Dynamics Dominate the Regulated Proteome during Early *Xenopus* Development

**DOI:** 10.1038/s41598-018-35442-z

**Published:** 2018-11-21

**Authors:** Elizabeth H. Peuchen, Olivia F. Cox, Liangliang Sun, Alex S. Hebert, Joshua J. Coon, Matthew M. Champion, Norman J. Dovichi, Paul W. Huber

**Affiliations:** 1 Department of Chemistry and Biochemistry, University of Notre Dame, Notre Dame, IN 46556 USA; 2 Department of Chemistry, Michigan State University, East Lansing, MI 48824 USA; 3 Department of Chemistry, University of Wisconsin, Madison, WI 53706 USA

Correction to: *Scientific Reports* 10.1038/s41598-017-15936-y, published online 15 November 2017

This Article contains an error in Figure 1, with the wrong heatmap in Figure 1D present. The correct Figure 1 appears below. The Figure legend is correct.

**Figure 1:**
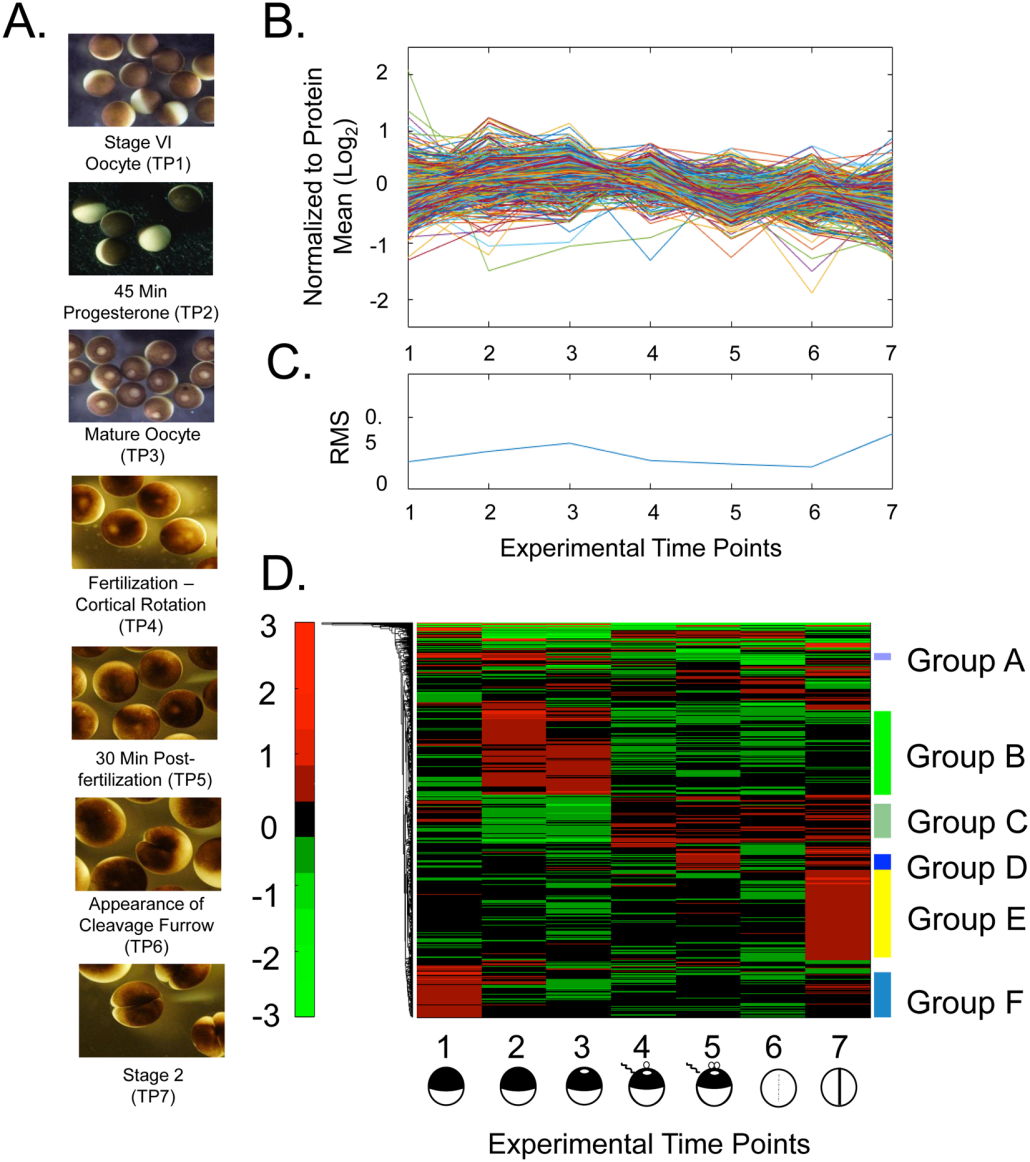
Quantitative changes of the *X. laevis* proteome over seven developmental stages. (**A**) Micrographs of *X. laevis* at the developmental stages taken for proteomic analyses. (**B**) Individual protein abundance presented on a log_2_ axis across developmental time points. Data are normalized to the mean value of each protein. (**C**) Relative standard deviation (RSD) for all proteins in panel (B) at individual experimental time points. (**D**) Heatmap showing the change in protein expression across the developmental time points. The heatmap was generated from the log_2_ normalized data using the default parameters in Matlab. Six groups representing significant trends of protein expression were manually selected for analysis. Proteins with the 15% greatest quantitative change are presented and are considered significant at a FDR of 0.20 using a Benjamini-Hochberg test. Neither clustering nor dendrogram generation was performed along the development stage axis of the heatmaps. These points are defined by the biology of development, and their differences are fixed by that biology.

