# Supplementary material for: Phosphorylation Dynamics Dominate the Regulated Proteome during Early Xenopus Development
Source: Sci Rep. 2017 Nov 15;7:15647. doi: 10.1038/s41598-017-15936-y (PMC5688136; doi:10.1038/s41598-017-15936-y)

Phosphorylation Dynamics Dominate the Regulated Proteome during Early *Xenopus* Development

Elizabeth H. Peuchen[1], Olivia F. Cox[1], Liangliang Sun[2], Alex S. Hebert[3], Joshua J. Coon[3], Matthew M. Champion[1], Norman J. Dovichi[1], Paul W. Huber[1*]

Affiliations:

1. Department of Chemistry and Biochemistry, University of Notre Dame, Notre Dame, IN 46556 U.S.A.
2. Department of Chemistry, Michigan State University, East Lansing, MI 48824 U.S.A.
3. Department of Chemistry, University of Wisconsin, Madison, WI 53706 U.S.A.

Table of Contents:

Figure S1. Protein change during the seven developmental time points.
Figure S2. Unpacked galaxy plot of the development.
Figure S3. Heat map of all phosphorylation site changes.
Figure S4. Kinase consensus sequence profiles.

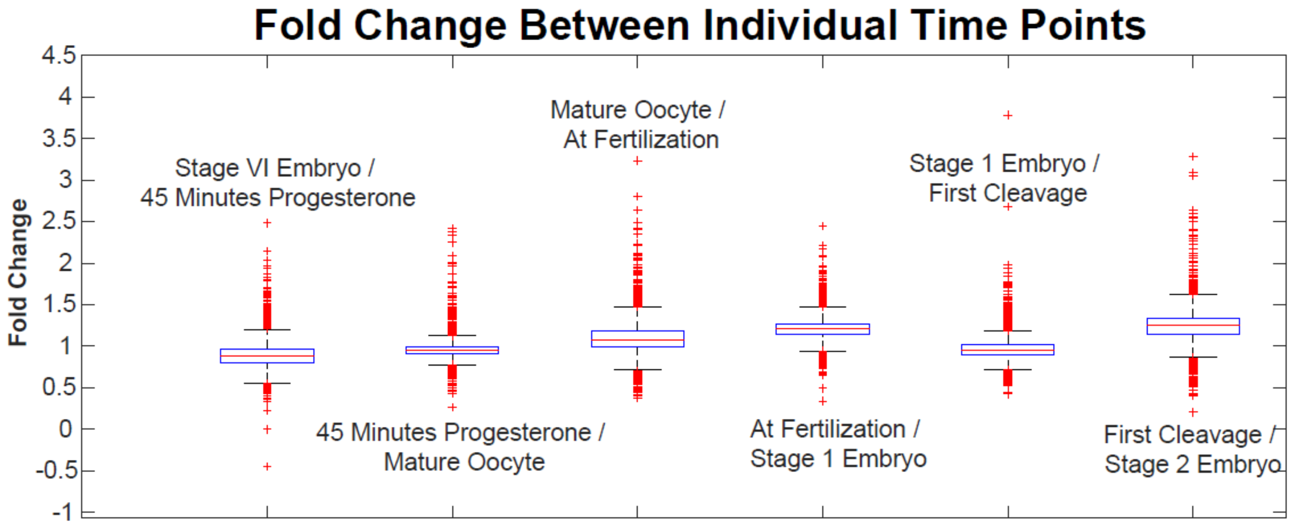

Figure S1. Protein change during the seven developmental time points. Related to Figure 1. Box and whisker plot demonstrating the fold change of the proteins relative to their adjacent stage. Outliers were determined by Matlab's default Box plot algorithms and are marked by the '+'. One outlier at 11.92 fold change was removed for the Stage VI / 45 minutes' progesterone sample for clarity of the figure.

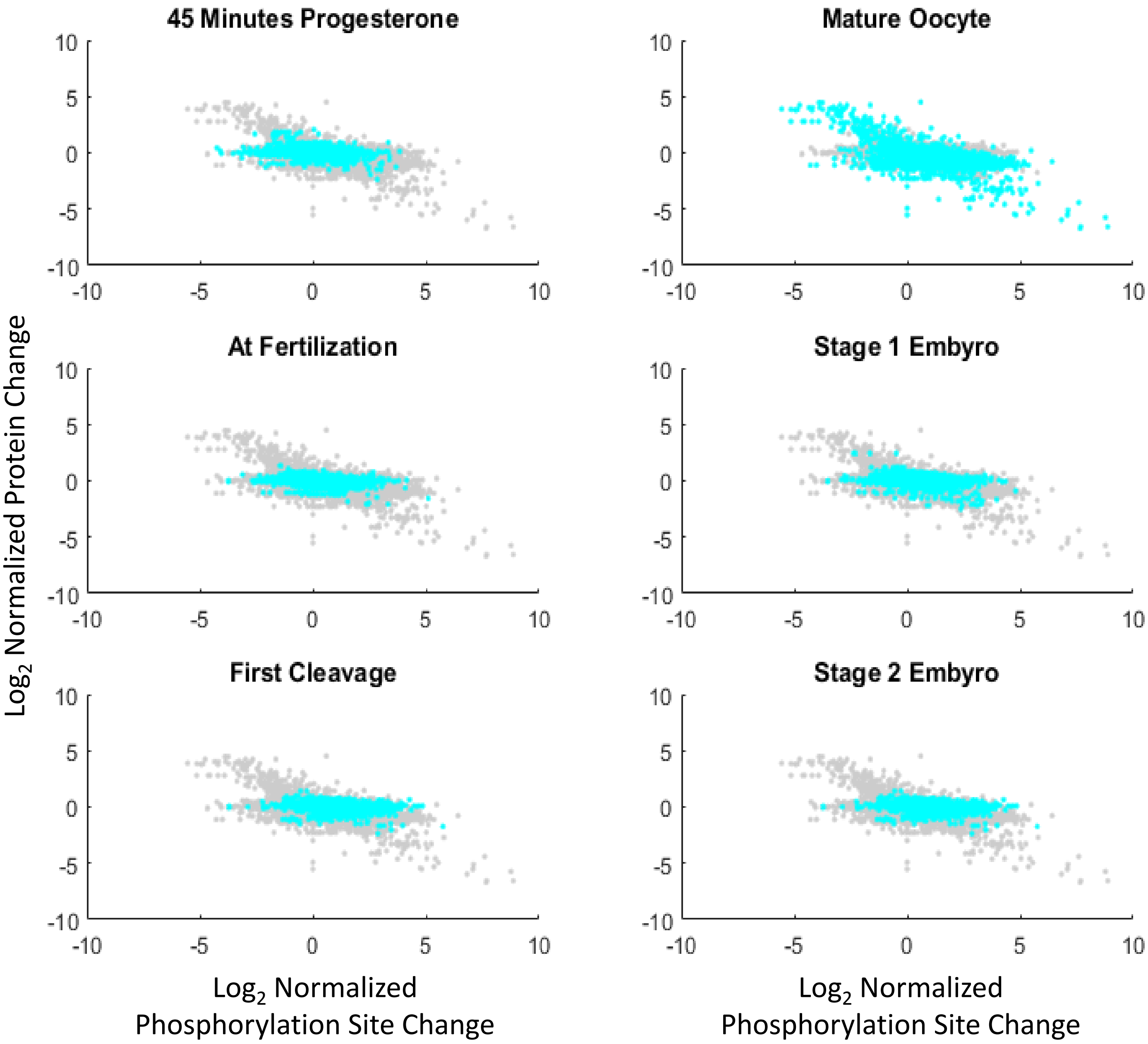

Figure S2. Unpacked galaxy plot of the development. Related to Figure 3. Each of the individual time points shown in relation to protein change. Figure 3 is a combination of the six galaxy plots overlaid in the manuscript. The grey background for each panel is of the other time points. The turquois blue describes the expression of the titled time point protein vs. phosphorylation site.

A

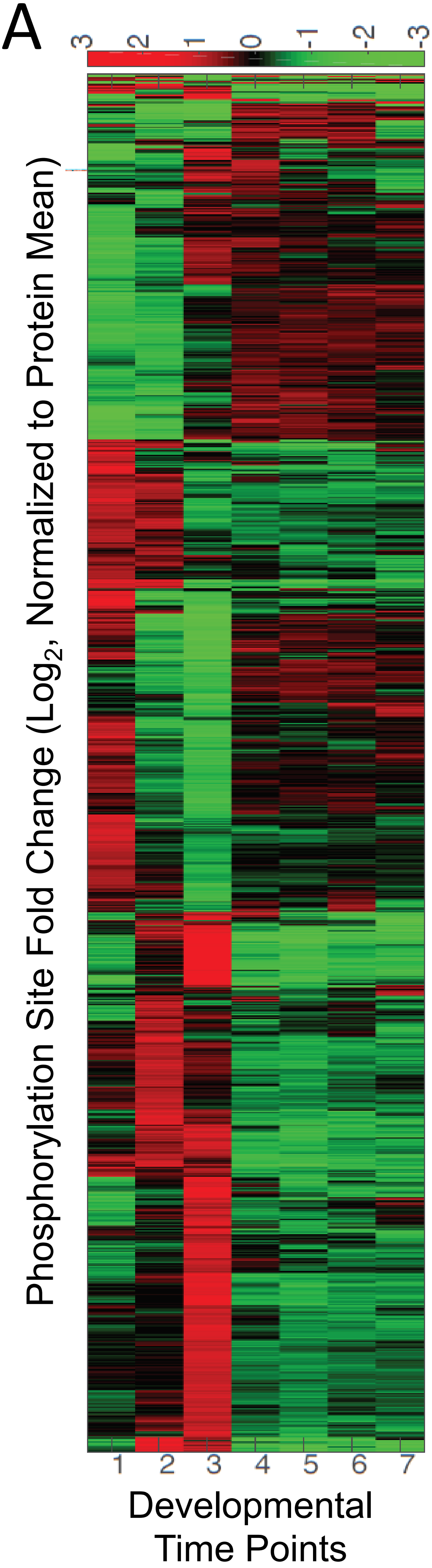

Figure S3. Heat map of all phosphorylation site changes. Related to Figure 4. (A) Full heat map of all phosphorylation sites corrected for their protein level. Figure 4 only includes the top 60% of phosphorylation site variance. Protein mean is determined based on the normalized signal intensity of all the proteins in the sample for the respective time point. **(B)** Cohen's D Analysis of individual phosphorylation sites between time points. A $\Delta \geq 0.25$ is considered significant:

$$Cohen's\ \Delta = \frac{(Mean\ 1 - Mean\ 2)}{Stdev\ (Mean\ 1\ x\ 2)}$$

B

Mean 2 (columns); **Mean 1** (rows)

	TP1	TP2	TP3	TP4	TP5	TP6	TP7
TP1	0.00	-0.72	-1.25	-0.76	-1.01	-0.07	-0.55
TP2	0.72	0.00	-0.90	0.13	-0.23	0.71	0.29
TP3	1.25	0.90	0.00	1.03	0.84	1.25	1.08
TP4	0.76	-0.13	-1.03	0.00	-0.45	0.77	0.22
TP5	1.01	0.23	-0.84	0.45	0.00	1.04	0.61
TP6	0.07	-0.71	-1.25	-0.77	-1.04	0.00	-0.53
TP7	0.55	-0.29	-1.08	-0.22	-0.61	0.53	0.00

Figure S4. Kinase consensus sequence profiles. Related to Figure 6. All kinase sequences and their respective amino acid sequence are presented here. Figure 6 shows cluster 4 and 20.

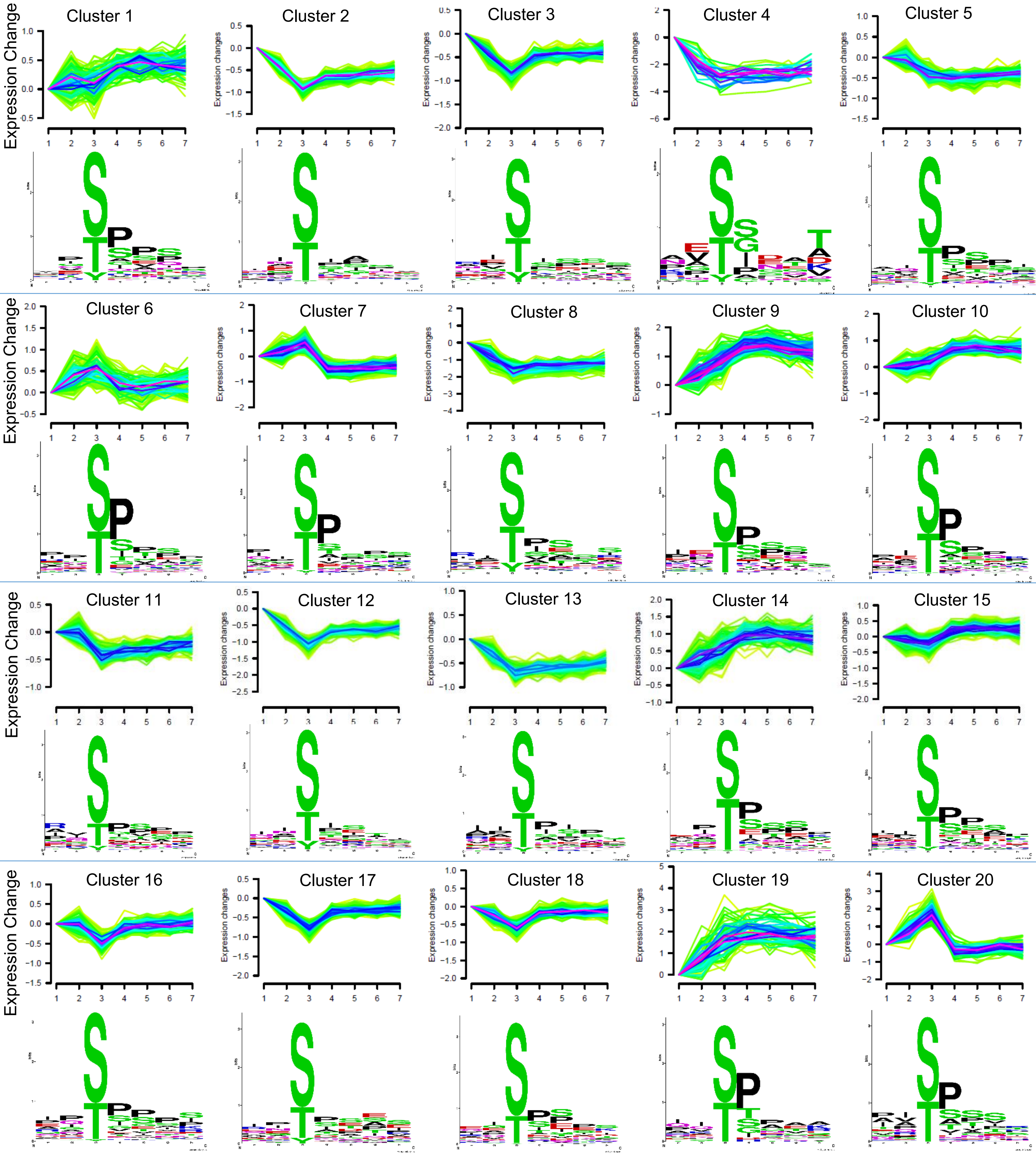

Figure S4 Continued.

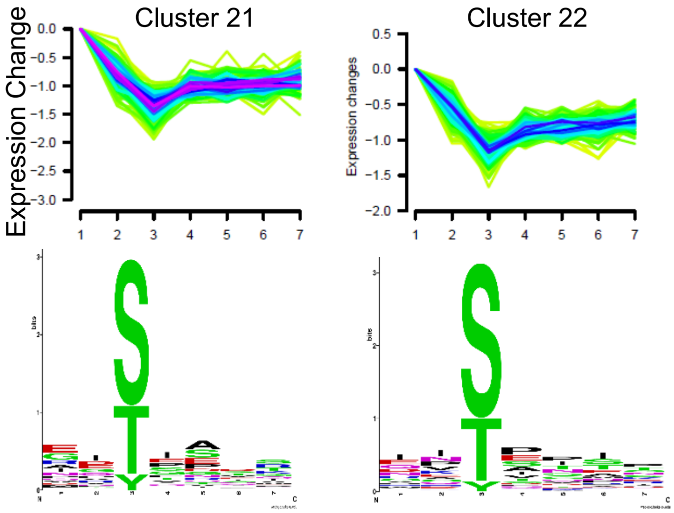

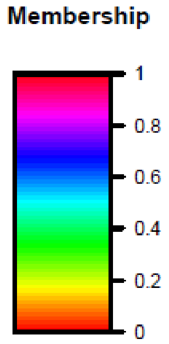

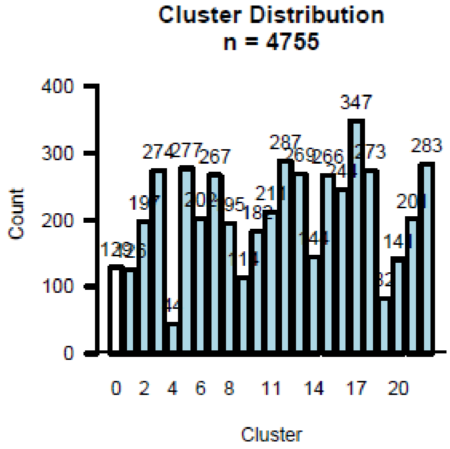

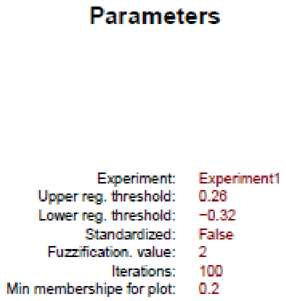

Supplement: Supplementary file 1 — Supplementary Figures [file 41598_2017_15936_MOESM1_ESM.pdf]
